# A Prognostic Model Based on Immune-Related Long Non-Coding RNAs for Patients With Cervical Cancer

**DOI:** 10.3389/fphar.2020.585255

**Published:** 2020-11-30

**Authors:** Peijie Chen, Yuting Gao, Si Ouyang, Li Wei, Min Zhou, Hua You, Yao Wang

**Affiliations:** Medical Oncology Department, Affiliated Cancer Hospital & Institute of Guangzhou Medical University, Guangdong, China

**Keywords:** Cervical cancer, Long non-coding RNA, Immunology, Gene express, Prognosis

## Abstract

**Objectives:** The study is performed to analyze the relationship between immune-related long non-coding RNAs (lncRNAs) and the prognosis of cervical cancer patients. We constructed a prognostic model and explored the immune characteristics of different risk groups.

**Methods:** We downloaded the gene expression profiles and clinical data of 227 patients from The Cancer Genome Atlas database and extracted immune-related lncRNAs. Cox regression analysis was used to pick out the predictive lncRNAs. The risk score of each patient was calculated based on the expression level of lncRNAs and regression coefficient (β), and a prognostic model was constructed. The overall survival (OS) of different risk groups was analyzed and compared by the Kaplan–Meier method. To analyze the distribution of immune-related genes in each group, principal component analysis and Gene set enrichment analysis were carried out. Estimation of STromal and Immune cells in MAlignant Tumors using Expression data was performed to explore the immune microenvironment.

**Results:** Patients were divided into training set and validation set. Five immune-related lncRNAs (H1FX-AS1, AL441992.1, USP30-AS1, AP001527.2, and AL031123.2) were selected for the construction of the prognostic model. Patients in the training set were divided into high-risk group with shorter OS and low-risk group with longer OS (*p* = 0.004); meanwhile, similar result were found in validation set (*p* = 0.013), combination set (*p* < 0.001) and patients with different tumor stages. This model was further confirmed in 56 cervical cancer tissues by Q-PCR. The distribution of immune-related genes was significantly different in each group. In addition, the immune score and the programmed death-ligand 1 expression of the low-risk group was higher.

**Conclusions:** The prognostic model based on immune-related lncRNAs could predict the prognosis and immune status of cervical cancer patients which is conducive to clinical prognosis judgment and individual treatment.

## Background

Cervical cancer is the fourth most common malignant tumor among women worldwide, both in morbidity and mortality (Bray et al., 2018). Approximately 90% of cervical cancer cases in recent years occurred in developing countries, with a higher rate of morbidity and mortality than in developed countries (WHO, 2018). Most of the early stage cervical cancer patients can be cured by surgery, and the primary treatment for locally advanced cervical cancer is chemo-radiotherapy. The 5-year overall survival (OS) rate of stage I cervical cancer patients is 92.1%, and that of stages II, III, and IV patients is 74.2, 52.0, and 29.8%, respectively (Nagase et al., 2019). While the drug resistance to patients leads to limited therapeutic response, including chemotherapeutics and radiation therapy; it has become a serious problem on cervical cancer therapy (Burger et al., 2011; Eskander and Tewari, 2014). With the advancement in tumor immunology, especially the development of immune checkpoint inhibitors (ICIs), the treatment of cervical cancer has made some progress in immunotherapy and targeted therapy (Menderes et al., 2016; De Felice et al., 2018). Evidence suggests that the objective response rate (ORR) of programmed death-ligand 1 (PD-L1) inhibitor is only about 14% (Chung et al., 2019). Nevertheless, the survival status and prognosis of recurrent and advanced cervical carcinoma patients is not yet satisfactory. Thus, performing risk stratification with immune factors for patients with cervical cancer may be helpful in predicting their survival and immunotherapeutic response.

Long non-coding RNA (lncRNA), a non-protein coding transcript, is more than 200 nucleotides in length. Previous research revealed that lncRNAs are extensively involved in different aspects of the immune system, including immune cell lineage development, immune cell activation, and immune-related diseases (Atianand et al., 2017). Notably, lncRNA is reported as a critical regulator in cancer immunity, covering antigen presentation, immune stimulation, tumor infiltration and so on (Yu et al., 2018). Numerous studies on cancer indicated that lncRNAs take part in tumorigenesis of cervical cancer, being closely correlated with the prognosis of patients. For instance, gastric carcinoma high expressed transcript 1 is considered a carcinogenic lncRNA that promotes proliferating, migrating and infiltrating in different kinds of cancer, including cervical cancer (Zhang et al., 2019). Another study demonstrated that lncRNA cancer susceptibility 15 plays a driving role in cell proliferation, invasion, cycle progression, and epithelial-to-mesenchymal signaling pathway of cervical cancer (Shan et al., 2019). LINC0051 also promote the progression of cervical cancer, as well as resistance to paclitaxel (Mao et al., 2019). Hence, lncRNA may act as a potential target in the treatment and prognosis of cervical cancer. However, we have not yet identified exactly what role the immune-related lncRNAs play in the prognosis of cervical cancer. Our study was, therefore, designed to explore the correlation between immune-related lncRNAs and the prognosis of cervical cancer, and construct a prognostic model by analyzing the gene expression profile in The Cancer Genome Atlas (TCGA) database.

## Materials and Methods

### Samples and Datasets

In this research, we downloaded the gene expression profiles of tumor samples, and the corresponding prognostic information of 255 cervical cancer patients from the TCGA database (https://cancergenome.nih.gov/). Patients were excluded if the survival time was ≤30 days because they may have died of other fatal complications. Finally, 227 patients were enrolled in our research and each sample corresponded to one patient. The combination set were divided into training set 167 and validation set 60 randomly for the following study. Data collection date was January 10, 2020.

### Immune-Related Long Non-Coding RNAs Extraction and Mining

Extraction and mining methods of immune-related lncRNAs were described previously (Wang et al., 2018; Wei et al., 2019). We obtained the immune-related genes from Molecular Signatures Database 4.0.1 (Immune system process M13664, Immune response M19817) on Gene Set Enrichment Analysis (GSEA) website (http://software.broadinstitute.org/gsea/index.jsp) (Wang et al., 2018). LncRNAs were extracted by the GENCODE project (http://www.gencodegenes.org) (Derrien et al., 2012). We obtained the expression levels of immune genes and lncRNAs in each sample, and the cohort of immune-related lncRNAs was identified according to Pearson’s correlation analysis by the cor. test function of R (correlation coefficient Cor > 0.6, *p* < 0.001).

### Prognostic Model Construction and Validation

The training set including 167 patients were used to construct the model. The Survival package of R (3.5.2) software was used for multivariate analysis of lncRNAs with statistically significant differences in univariate analysis, and the optimal prediction model was determined based on the Akaike Information Criterion (AIC). The risk score of each patient was determined by the lncRNAs’ expression level and the regression coefficient (*β*) of the weighted linear combination in the multivariate analysis. The formula was listed as follows: Risk score = *β* gene 1 × expr (gene 1) + β gene 2 × expr (gene 2) + …+ β gene N × expr (gene N). exprgene referred to the expression of lncRNAs. According to the median risk score, we divided all the patients into two groups: high-risk group and low-risk group. To evaluate the accuracy of this prognostic model, the same algorithm was performed in the validation set (60 patients) and combination set (227 patients) with the same coefficient (*β*), and also performed for further examination of cancer tissues from 56 cervical cancer patients in our center.

### Patient Eligibility and Evaluation

We enrolled 56 cervical cancer patients treated in Affiliated Cancer Hospital & Institute of Guangzhou Medical University between January 1, 2013 and December 30, 2017. All of the patients were diagnosed with postoperative histopathological examination. Primary cancer tissues were stored in RNA*later*™ Stabilization Solution (Invitrogen) immediately after resection at −80 °C. This study was approved by the Ethic Committee of Affiliated Cancer Hospital & Institute of Guangzhou Medical University, and written informed consent was obtained from each patient. Baseline characteristics were obtained from the patients’ history. OSs were measured from the date of diagnosis to either the end of the follow-up period, to the date of death from any cause or to the date of loss to follow-up.

### Quantitative Real-Time PCR

Total RNA was extracted from tissue samples using TRIzol (Invitrogen) according to the manufacturer’s protocol. Samples were treated with DNase using the RNase-free DNase Set (Qiagen) during the total RNA isolation. First strand complementary DNA (cDNA) was synthesized using the cDNA Synthesis kit (Thermo Fisher Scientific) according to the manufacturer’s instructions. ABI prism 7900-HT sequence detection system (96-well, Applied Biosystems) was used to perform quantitative real-time PCR (RT-PCR) analysis. For RT-PCR, the following primers were used:

GAPDH: 5′- GAC​TCA​TGA​CCA​CAG​TCC​ATG​C-3′ (forward), 5′- AGA​GGC​AGG​GAT​GAT​GTT​CTG-3′ (reverse).

H1FX-AS1: 5′- GAT​GGG​GAA​GGG​ATT​CGC​TC-3′ (forward), 5′- TCT​CCT​TTG​CTG​TGT​TCC​CG-3′ (reverse).

AL441992.1: 5′- AAG​AAG​CTC​TCG​TGT​GGC​TC-3′ (forward), 5′- TGG​CTT​TGA​AGC​GAG​GAT​GA-3′ (reverse).

USP30-AS1: 5′- AGC​AAT​AGC​TGA​CGG​ACC​AC-3′ (forward), 5′- TGA​AAA​CCA​AGC​AGC​CCC​A-3′ (reverse).

AP001527.2: 5′- ATT​GGG​AAT​GAC​TCA​TCT​GTT​TG-3′ (forward), 5′- AGC​AGT​AGA​CTC​CCA​GGA​AAG-3′ (reverse).

AL031123.2: 5′- ACA​CAC​GTG​GTC​TGT​AGC​G-3′ (forward), 5′- GGG​CCT​TGC​TTT​CCC​CAT​AA-3′ (reverse).

All samples were processed in triplicate. The relative gene expression was determined using the 2^−ΔΔCT^ method.

### Tumor Component Assessment

The distribution of immune-related genes was presented by principal component analysis (PCA). To identify whether the functional phenotypes were different between the high- and low-risk groups, GSEA was performed. Estimation of STromal and Immune cells in MAlignant Tumors using Expression data (ESTIMATE) was performed to evaluate the immune microenvironment, including the presence of stromal cell, tumor infiltration, and tumor purity in each sample (Yoshihara et al., 2013).

### Statistical Analysis

Kaplan–Meier curves were drawn to evaluate the OS and the data was statistically compared with the log rank test. The prognostic value of the immune-related lncRNAs was assessed by the univariate and multivariate cox proportional-hazards regression model. The receiver operating characteristic (ROC) curve was established to evaluate the reliability and accuracy of the prognostic model. All the statistical analyses were done using R software (version 3.5.2). *p* value (two-sided) < 0.05 was taken as being statistically significant.

## Results

### Patient Characteristics

We enrolled 227 patients with cervical cancer in our study, with an average age of 48.35 years (20–88 years) and sorted out their clinicopathological characteristics (Table 1).

**TABLE 1:** Characteristics of 227 patients with cervical cancer from The Cancer Genome Altas database.

Characteristics	N (%)
Age	
<60	178 (78.4)
≥60	49 (21.6)
Grade	
G1	10 (4.4)
G2	102 (44.9)
G3	92 (40.5)
G4	1 (0.4)
GX	20 (8.8)
unknown	2 (0.8)
FIGO stage	
I	101 (44.5)
II	50 (22.0)
III	13 (5.7)
IV	14 (6.2)
unknown	49 (21.6)
Tumor	
T1	101 (44.5)
T2	53 (23.3)
T3	14 (6.2)
T4	9 (4.0)
Tis	1 (0.4)
TX	14 (6.2)
unknown	35 (15.4)
Lymph node	
N0	93 (41.0)
N1	45 (19.8)
NX	54 (23.8)
unknown	35 (15.4)
Metastasis	
M0	88 (38.8)
M1	8 (3.5)
MX	94 (41.4)
unknown	37 (16.3)

### Identification of Immune-Related Long Non-Coding RNAs With Prognostic Value

We extracted 331 immune-related protein-coding genes from Molecular Signatures Database and obtained 121 immune-related lncRNAs by the co-expression network (correlation coefficient Cor > 0.6, *p* < 0.001). Finally, univariate Cox regression analysis based on the training set revealed nine immune-related lncRNAs with the most significant prognostic value for cervical cancer patients (Table 2). Among all the lncRNAs, AC015922.2 and AP001527.2 were considered as perilous factors while the rest were protective factors.

**TABLE 2:** Immune-related LncRNAs with significant prognostic value identified by univariate Cox regression analysis.

LncRNA	HR	*p* value
AL133215.2	0.413	0.017
H1FX-AS1	0.416	0.012
AC015922.2	1.306	0.025
AC097468.3	0.445	0.007
AL441992.1	0.600	0.019
USP30-AS1	0.656	0.006
AP001527.2	1.359	0.012
AL031123.2	0.382	0.013
AC024060.1	0.557	0.008

LncRNA, Long non-coding RNA.

### Construction and Verification of Prognostic Model

**FIGURE 1:**
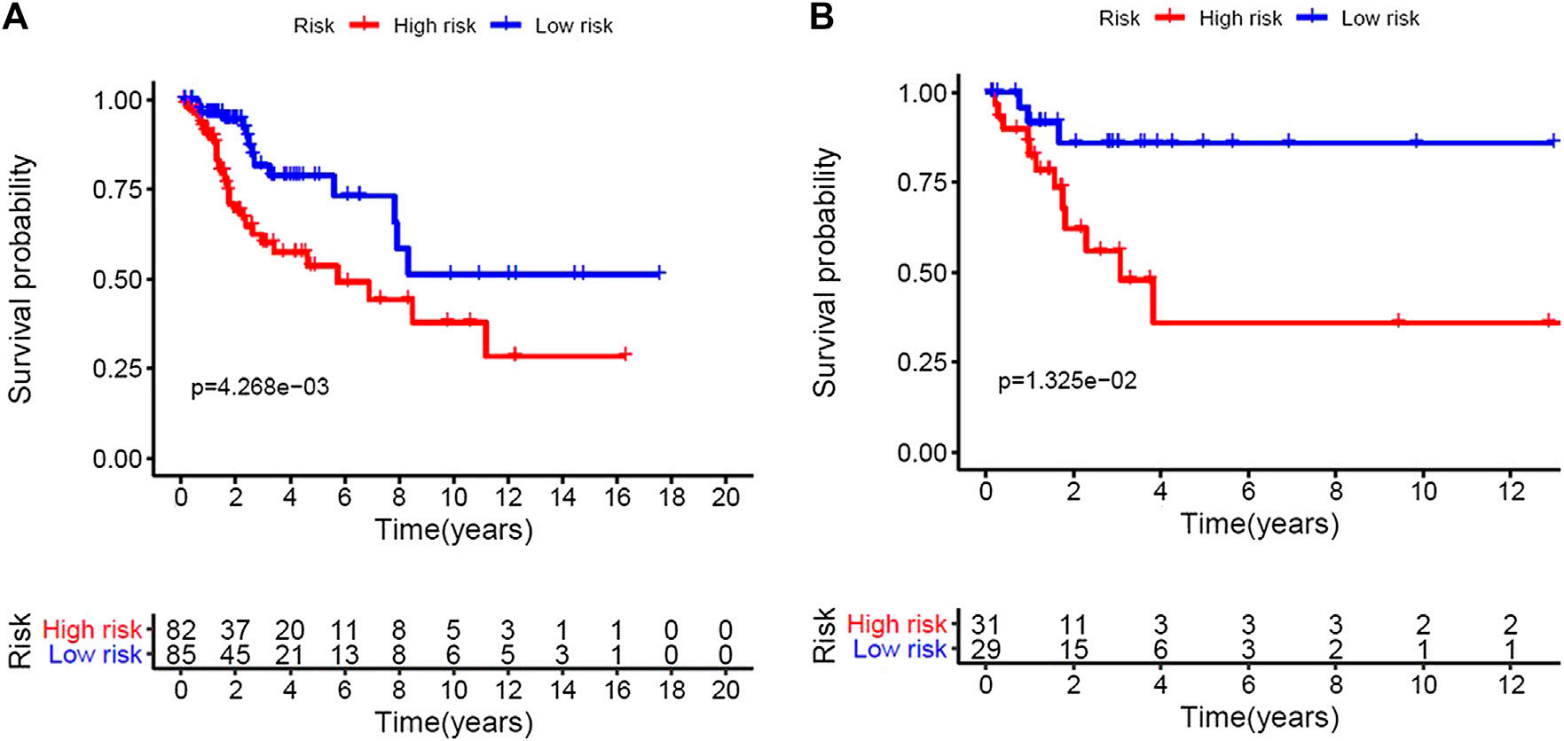
Construction of the five immune-related Long non-coding RNAs prognostic model of cervical cancer patients. Based on the median risk score, we divided patients from the training set into high-risk group with 84 individuals and low-risk group with 83 individuals. **(A)**, Kaplan–Meier curve of the high-risk and low-risk groups of training set showed differences in survival rate. **(B)**, Kaplan–Meier curve of the high-risk and low-risk groups of validation set also showed differences in survival rate.

**FIGURE 2:**
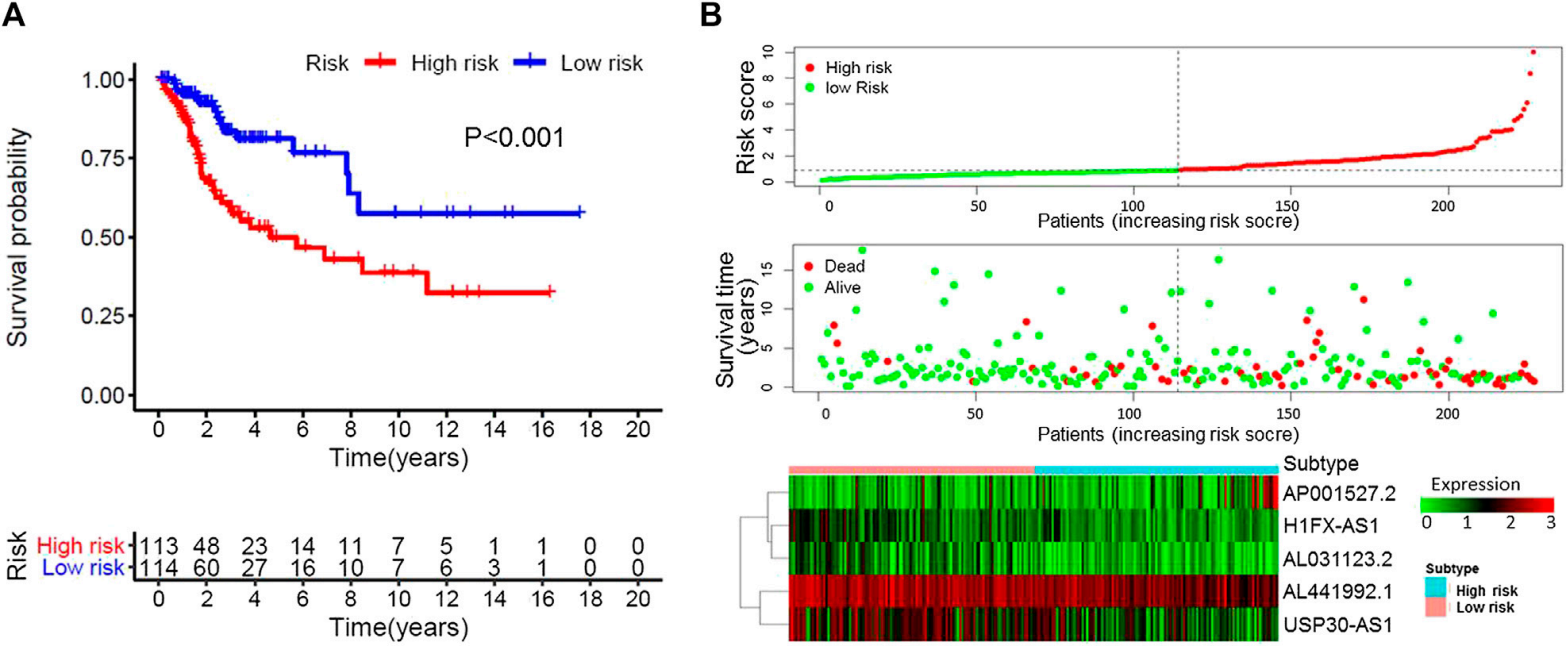
Characteristics of the patients in the combination set based on the constructed model. **(A)**, Kaplan–Meier curve of the high-risk and low-risk groups of combination set showed differences in survival rate. **(B)**, Distribution of risk score, survival status of each patients, and heat map expression of five immune-related Long non-coding RNAs in high-risk and low-risk groups were presented.

**FIGURE 3:**
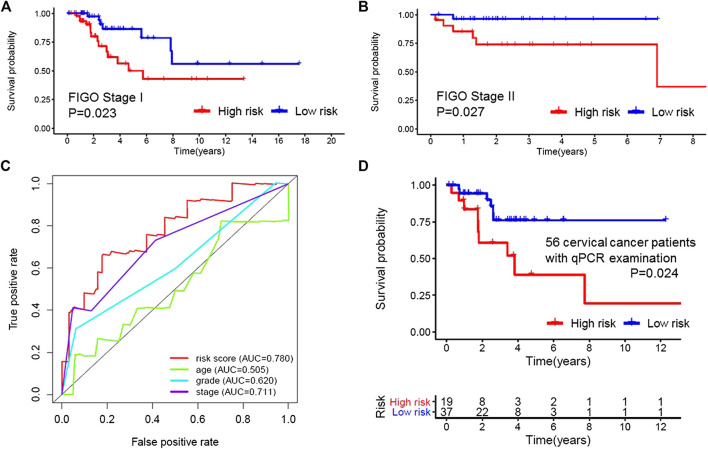
Applicability and reliability of the prognostic model. **(A,B)** Kaplan–Meier curve of FIGO stages I and II of the high- and low-risk groups. **(C)**, Receiver operating characteristic (ROC) curve of the risk score, age, grade, and stage. The area under the ROC curve (AUC) of the prediction model was 0.780, which was much better than that of age (0.505), grade (0.620) and FIGO stage (0.711). **(D)** Kaplan–Meier curve of 56 cervical cancer patients. 56 patients from our cancer center were selected and QRT-PCR was used to calculate the expression level of five Long non-coding RNAs, finally the prognostic model was validated using their accordingly clinical data.

Based on the multivariate analysis and the AIC value, five lncRNAs were used to construct the prognostic model (Table 3). The expression level of lncRNAs and regression coefficient (*β*) were integrated to calculate the risk score for each patient. Based on the median risk score, we divided the patients from the training set into a high-risk group with 84 individuals and a low-risk group with 83 individuals. Kaplan-Meier plot showed differences in survival rate between the two groups (*p* = 0.004, Figure 1A). We verified this model in the validation set (*p* = 0.013, Figure 1B) and combination set (*p* < 0.001, Figure 2A) with the similar result. In the combination set, the risk score and survival time of each risk group and the expression of five lncRNAs are shown in Figure 2B. To further investigate the value of this prognostic model in stratifying patients with different TNM stages, we carried out Kaplan-Meier analysis and showed that the risk subgroups differed significantly in both FIGO stage I and II (*p* = 0.023 and *p* = 0.027, respectively; Figures 3A,B). The area under the ROC curve (AUC) of the predictionmodel was 0.780, which was much better than that of age (0.505), grade (0.620), and FIGO stage (0.711) (Figure 3C). Moreover, 56 patients (Table 4) with cervical cancer were selected and QRTPCR was used to calculate the expression level of five lncRNAs, finally the prognostic modelwas validated using their accordingly clinical data. We found differences in survival rate between the two groups (p 0.024, Figure 3D).

**TABLE 3:** The optimal immune-related prognostic LncRNAs screened out by multivariate cox regression analysis and the AIC value, five lncRNAs were used to construct the prognostic model.

LncRNA	β	HR	*p* value
H1FX-AS1	−0.70	0.50	0.040
AL441992.1	−0.61	0.54	0.003
USP30-AS1	−0.28	0.76	0.045
AP001527.2	0.35	1.42	0.004
AL031123.2	−0.77	0.46	0.043

LncRNA, Long non-coding RNA.

**TABLE 4:** Characteristics of 56 patients with cervical cancer, whom were enrolled from our cancer center for further q-PCR examination.

Characteristics	N (%)
Age	
<60	40 (71.4)
≥60	16 (28.6)
Grade	
G1	7 (12.5)
G2	29 (51.8)
G3	20 (35.7)
FIGO stage	
I	17 (30.4)
II	25 (44.6)
III	9 (16.1)
IV	5 (8.9)
Death	
Yes	15 (26.8)
No	41 (73.2)
Risk group	
High	19 (33.9)
Low	37 (66.1)

### Immune Characteristics of High-Risk and Low-Risk Groups

**FIGURE 4:**
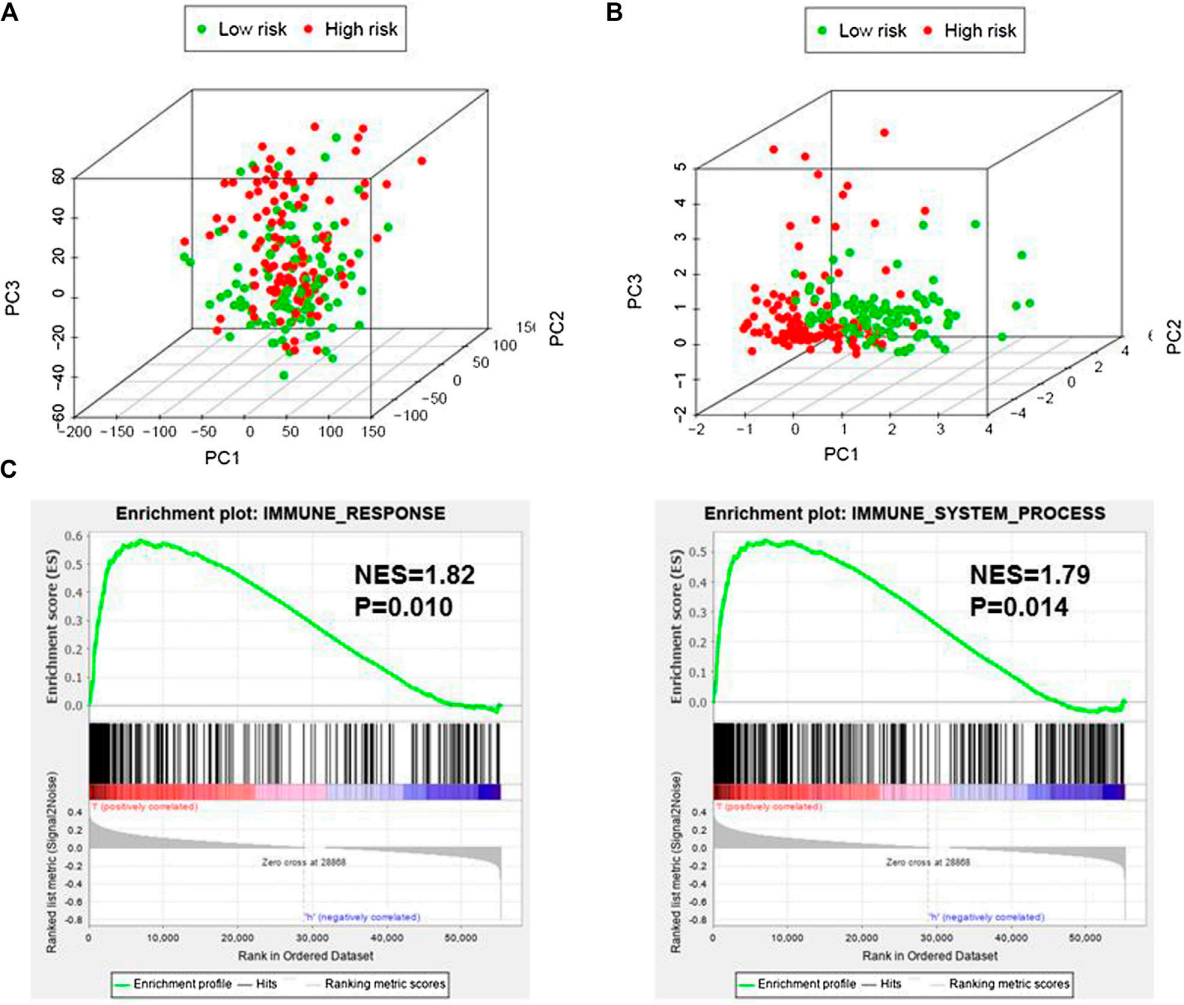
Immune status of the high- and low-risk groups. **(A)**, Principal component analysis (PCA) showed that whole gene expression profiles between the high- and low-risk groups were mixed up. **(B)**, PCA indicated that the distribution of immune-related genes between the high- and low-risk groups were significantly different. **(C)**, Gene set enrichment analysis implied that the low-risk group had adequate immune response and immune system process pathways. NES, Normalized Enrichment Score.

**FIGURE 5:**
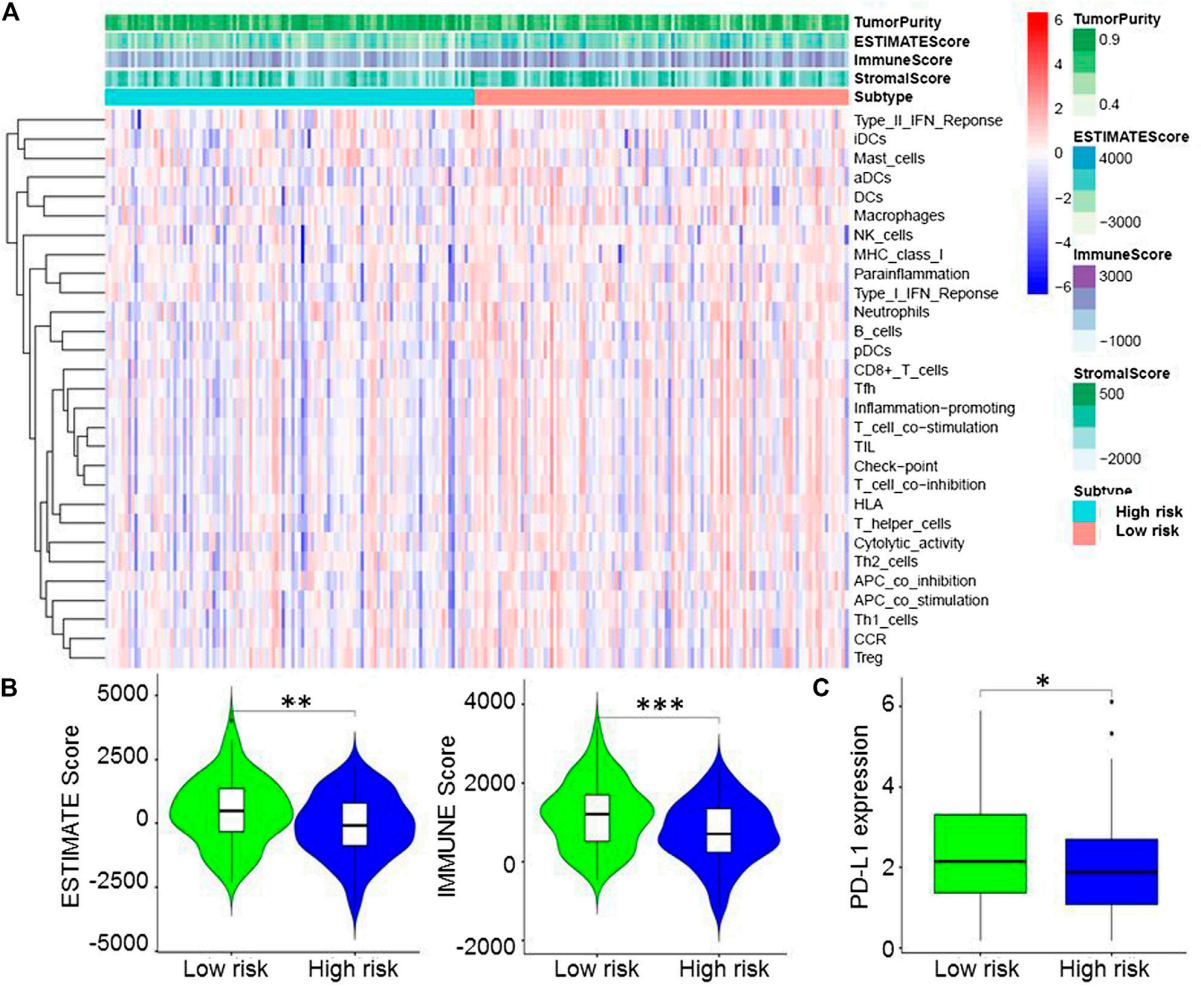
Immune microenvironment of the high- and low-risk groups. **(A)**, Estimation of STromal and Immune cells in MAlignant Tumors using Expression data (ESTIMATE) analysis showed that the immune score of the low-risk group was higher than that of the high-risk group. **(B)**, ESTIMATE analysis indicated that the low-risk group had more immune and stromal cells but lower tumor purity. **(C)**, Programmed death-ligand 1 expression of the low-risk group was higher than that of the high-risk group.

Based on immune genes and whole gene expression profiles, we investigated the distribution mode of the high-risk and low-risk groups by PCA in the combination set. On whole gene expression profiles, PCA showed that the high- and low-risk groups were mixed up (Figure 4A). While based on the immune genes, these two groups were obviously different, indicating that the distribution of immune-related genes between the high- and low-risk groups was significantly different (Figure 4B). Further analysis by GSEA showed that the low-risk group had adequate immune response and immune system process pathways (Figure 4C). According to the ESTIMATE analysis, the immune score of the low-risk group was higher than that of the high-risk group (Figure 5A). The low-risk group had more immune and stromal cells but lower tumor purity (Figure 5B). Meanwhile, PD-L1 expression of the low-risk group was higher than that of the high-risk group (Figure 5C), presenting a potential target for immunotherapy. Moreover, GO and KEGG enrichment analysis found that the functions of this group were mainly concentrated in immune-related functions (Table 5).

**TABLE 5:** Significantly enriched GO terms and KEGG pathways of low risk group.

GO	ID	Description	NSE	*p*
BP	GO:0002704	Regulation of leukocyte mediated immunity	2.14	0.015
BP	GO:0032480	Regulation of type I interferon production	2.12	0.014
BP	GO:0002707	Regulation of lymphocyte mediated immunity	2.10	0.014
BP	GO:0002710	Regulation of T cell mediated immunity	2.08	0.016
BP	GO:0031312	Extrinsic component of organelle membrane	2.06	0.022
KEGG	hsa04623	Cytosolic DNA sensing pathway	1.93	0.002
KEGG	hsa00190	Oxidative phosphorylation	1.89	0.006
KEGG	hsa05322	Systemic lupus erythematosus	1.87	0.002
KEGG	hsa05320	Autoimmune thyroid disease	1.87	0.002
KEGG	hsa04612	Antigen processing and presentation	1.80	0.012

BP, biological process; CC, cellular component; GO, Gene Ontology; MF, molecular function.

## Discussion

It has been reported that tumorigenesis is strongly associated with a series of cumulative genetic and epigenetic changes occurring in a normal cell; it is also closely related to the body’s microenvironment and immunity (Prendergast and Jaffee, 2007). The immune system recognizes and kills cancerous cells and their precursors, while cancerous cells develop strategies to escape from immune-surveillance thereby promoting tumorigenesis (Tesniere et al., 2006). Recently, lncRNA was proven to play an active part in the regulation of the immune system by affecting tumor microenvironment, epithelial-mesenchymal transition, dendritic cell and myeloid-derived stem cell regulation, and T and B cell activation and differentiation (Heward and Lindsay, 2014; Yu et al., 2015; Denaro et al., 2019). Immune-related lncRNAs, which were identified as a prognostic marker of various types of cancer (Wang et al., 2018), are markedly connected with immune cell infiltration (Li et al., 2020), and might be a potential target for cancer treatment.

Many lncRNAs have been shown to participate in the occurrence and progression of cervical cancer, either promoting (like SNHG7) or inhibiting (like GAS5) the disease (Cao et al., 2014; Zeng et al., 2019). Recent study identified a two-lncRNA signature (ILF3-AS1 and RASA4CP) as an independent biomarker which could predict the prognosis of cervical cancer based on the TCGA database and quantitative reverse transcription PCR (qRT-PCR) (Wu et al., 2019). This new finding further proved the importance of lncRNAs in cervical cancer, enlarged its application prospect on the prognosis of the disease, and emphasized the significance of future study on the function and mechanism of lncRNAs. In this study, we explored the connection between immune-related lncRNAs and prognosis of cervical cancer. Among the five immune-related lncRNAs used to construct the model, H1FX-AS1, AL441992.1, USP30-AS1, and AL031123.2 were protective factors, while AP001527.2 was a risk factor. As far as we know, all of these five immune-related lncRNAs have not been studied in clinical and fundamental research. We hypothesized that the immune-related lncRNAs in our study might play a similar role in cervical cancer.

The prognostic model demonstrated superior ability in dividing patients into low- and high-risk groups. We found that patients in the low-risk group showed favourable prognosis, either the training set or the validation set, as well as in the combination set, which indicate that our model might be capable of risk stratification. The AUC for the prognostic model was 0.780, which was greater than the AUC of other clinicopathological factors. Moreover, this model can also distinguish the prognosis of patients in FIGO stages I and II, and could be an important supplement to FIGO stage. It is important that the value of the prognostic model was further verified with the tissue samples of patients.

There is evidence demonstrating that immunotherapy is a novel therapeutic strategy for cervical cancer treatment (Eskander and Tewari, 2015; Ventriglia et al., 2017). While the efficacy of immunotherapy varies from person to person, the ORR of nivolumab (ICI, anti-PD-1) in cervical cancer is 26.3% and the median OS is 21.9 months (Naumann et al., 2019). The efficiency of immunotherapy depends on the immunogenicity of the tumor microenvironment, therefore knowing more about the tumor microenvironment is the key to evaluating the probability of immunotherapy (Gasser et al., 2017). The predictive biomarkers of cancer immunotherapy mainly include PD-L1 expression, immune cell infiltration, tumor mutational burden, specific gene mutations, and so on (Chen et al., 2018; Darvin et al., 2018; Yi et al., 2018; Otoshi et al., 2019). Though the efficacy of immunotherapy is better than that for traditional treatment, only a few patients are suitable for it because of limited availability and high cost. It is essential to screen the appropriate individuals for immunotherapy. We found that the distribution of immune-related genes was significantly different in each group, the immune score and the PD-L1 expression of the low-risk group was higher, GSEA revealed that the low-risk group had abundant immune response and immune system process pathways. Thus, the low-risk group might be more suitable for immunotherapy because of possessing high immunogenicity but the mechanism remains unclear. Moreover, we ultimately look forward to verify the response rate of ICIs in different risk group, further work is needed to validate these findings in future.

## Conclusion

We constructed a prognostic model based on immune-related lncRNAs to evaluate the prognosis of patients with cervical cancer, the high- and low-risk groups displayed different immune states, indicating that immunogenicity might be a potential factor to determine the suitability of patients for immunotherapy. We expect this model to be helpful in clinical treatment, but its application value needs to be further verified by a multicenter, large-sample clinical study.

## Data Availability

The original contributions presented in the study are included in the article/supplementary material, further inquiries can be directed to the corresponding author/s.
