# Corrigendum: A Prognostic Model Based on Immune-Related Long Non-Coding RNAs for Patients With Cervical Cancer

**DOI:** 10.3389/fphar.2021.723153

**Published:** 2021-08-25

**Authors:** Peijie Chen, Yuting Gao, Si Ouyang, Li Wei, Min Zhou, Hua You, Yao Wang

**Affiliations:** Medical Oncology Department, Affiliated Cancer Hospital and Institute of Guangzhou Medical University, Guangdong, China

**Keywords:** gene express, cervical cancer, long non-coding RNA, immunology, prognosis

In the original article, there were several errors. In the Abstract longer and shorter were switched when discussing the prognosis of the high-risk and low-risk group. A correction has been made to the Abstract, Paragraph 3:

“Results: Patients were divided into training set and validation set. Five immune-related lncRNAs (H1FX-AS1, AL441992.1, USP30-AS1, AP001527.2, and AL031123.2) were selected for the construction of the prognostic model. Patients in the training set were divided into high-risk group with shorter OS and low-risk group with longer OS (*p* = 0.004); meanwhile, similar result were found in validation set (*p* = 0.013), combination set (*p* < 0.001) and patients with different tumor stages. This model was further confirmed in 56 cervical cancer tissues by Q-PCR. The distribution of immune-related genes was significantly different in each group. In addition, the immune score and the programmed death-ligand 1 expression of the low-risk group was higher.”

In addition, **Figure 1A** was cited where **Figure 1B** should have been and the *p* value of **Figure 3B** was incorrectly stated as 0.032 when it should be 0.027. A correction has been made to the **Results** section, subsection **Construction and Verification of Prognostic Model**:

“Based on the multivariate analysis and the AIC value, five lncRNAs were used to construct the prognostic model (**Table 3**). The expression level of lncRNAs and regression coefficient (*β*) were integrated to calculate the risk score for each patient. Based on the median risk score, we divided the patients from the training set into a high-risk group with 84 individuals and a low-risk group with 83 individuals. Kaplan-Meier plot showed differences in survival rate between the two groups (*p* = 0.004, **Figure 1A**). We verified this model in the validation set (*p* = 0.013, **Figure 1B**) and combination set (*p* < 0.001, **Figure 2A**) with the similar result. In the combination set, the risk score and survival time of each risk group and the expression of five lncRNAs are shown in **Figure 2B**. To further investigate the value of this prognostic model in stratifying patients with different TNM stages, we carried out Kaplan-Meier analysis and showed that the risk subgroups differed significantly in both FIGO stage I and II (*p* = 0.023 and *p* = 0.027, respectively; **Figures 3A,B**). The area under the ROC curve (AUC) of the prediction model was 0.780, which was much better than that of age (0.505), grade (0.620), and FIGO stage (0.711) (**Figure 3C**). Moreover, 56 patients (**Table 4**) with cervical cancer were selected and QRT-PCR was used to calculate the expression level of five lncRNAs, finally the prognostic model was validated using their accordingly clinical data. We found differences in survival rate between the two groups (*p* = 0.024, **Figure 3D**).”

The authors apologize for this error and state that this does not change the scientific conclusions of the article in any way. The original article has been updated.

